# The association between advanced glycation end products (AGEs) and ABC (hemoglobin A1C, blood pressure, and low-density lipoprotein cholesterol) control parameters among patients with type 2 diabetes mellitus

**DOI:** 10.1186/s13098-022-00895-w

**Published:** 2022-08-26

**Authors:** Mohaddeseh Rezaei, Soghra Rabizadeh, Maryam Mirahmad, Minoo Sadat Hajmiri, Manouchehr Nakhjavani, Mahboobeh Hemmatabadi, Nooshin Shirzad

**Affiliations:** 1 Endocrinology and Metabolism Research Center, Endocrinology and Metabolism Clinical Sciences Institute, Tehran University of Medical Sciences, Tehran, Iran; 2 Department of Endocrinology, Endocrinology and Metabolism Research Center (EMRC), Vali-Asr Hospital, Imam Khomeini Hospital Complex, Tehran University of Medical Sciences, Tehran, Iran; 3 Department of Endocrinology, Hamadan University of Medical Sciences, Hamadan, Iran

**Keywords:** Type 2 diabetes, Glycemic control, ABC goals, Advanced glycation end-products, Diabetes mellitus, Disease management

## Abstract

**Background:**

Diabetes-induced chronic hyperglycemia results in the formation and aggregation of advanced glycation end-products (AGEs), which are products of non-enzymatic glycosylation of lipids or proteins. The development of diabetic complications can be accelerated by AGEs. In the current study, we aimed to explore the relationship between AGEs levels and ABC goals of diabetes control (A: Hemoglobin A1C < 7.0%, B: Blood pressure < 140/90 mmHg, and C: low-density lipoprotein cholesterol [LDL] < 100 mg/dL).

**Methods:**

In the current cross-sectional study, 293 patients with type 2 diabetes mellitus (T2D), were enrolled. Demographic and clinical characteristics of the individuals were collected. AGEs levels were measured using quantitative fluorescence spectroscopy. Finally, the association of AGEs levels with patients' characteristics and ABC goals was assessed.

**Results:**

Higher serum AGEs concentration was detected in older age, smoking patients and those with higher diastolic blood pressure, lower high-density lipoprotein (HDL) level, lower body mass index (BMI) and retinopathy. Moreover, the T2D patients who achieved higher numbers of ABC goals of diabetes were younger age (P-value = 0.003), with lower hemoglobin A1C (P-value = 0.001), fasting blood sugar (P-value = 0.002) diastolic blood pressure (P-value = 0.001), systolic blood pressure (P-value = 0.001), cholesterol (P-value = 0.001), LDL (P-value = 0.001), and AGEs (P-value = 0.023) levels. Diabetic patients with AGEs levels above 73.9% were about 2.2 times more likely to achieve none of ABC treatment goals (95% CI 1.107–3.616).

**Conclusion:**

Our results revealed the relationship between AGEs and ABC goal achievement, and microvascular diabetic complications, and imply that AGEs measurement may be valuable in the monitoring of diabetic patients' complications and treatment adjustment.

## Background

According to the statistics from the International Diabetes Federation, the global population of people aged 20–79 years living with diabetes is estimated to be around 537 million cases. Furthermore, the number of adult diabetic individuals is projected to increase in the future decades as a result of industrialization, aging, and changes in physical activity and diet. By 2045, the total number of patients with diabetes is expected to reach about 783 million [1].

Approximately 90% of people with diabetes suffer from type 2 diabetes mellitus (T2D). The increasing number of undiagnosed patients and high prevalence of diabetes mellitus has led to a costly rise in the occurrence of diabetic complications. The complications of diabetes mellitus are categorized into microvascular complications such as retinopathy, nephropathy, and neuropathy, as well as macrovascular complications (e.g., cardiovascular disease [CVD]) [2].

Intensive control of blood glucose [3], Lipid profile [4], and blood pressure (BP) [5] can help to lower the risk of diabetic complications. Adults with diabetes should maintain defined ABC goals (A: hemoglobin A1C [HbA1C] < 7.0%, B: BP < 140/90 mmHg, and C: low-density lipoprotein cholesterol [LDL] < 100 mg/dL), according to American Diabetes Association guidelines [6].

Chronic hyperglycemia caused by diabetes results in the aggregation of non-enzymatically derived glycosylation products of lipids and proteins. Advanced glycation end-products (AGEs) contribute to the pathogenesis of chronic diabetic complications [7]. AGEs are comprised of a heterogeneous set of complex molecules that are produced by non-enzymatic reactions involving free amino groups on nucleic acids, proteins, or lipids and carbonyl groups of reducing sugars. In individuals with diabetes, the production and accumulation of AGEs, which are natural aging processes driven by hyperglycemia, occurs at a faster rate [8].

RAGE is produced as a result of intrinsic cellular signaling that is induced by AGEs. AGEs and their receptors (RAGE) are thought to be crucial components of the inflammatory processes that might lead to diabetes and its complications [9]. Furthermore, early hyperglycemia is thought to cause a proportionate rise in AGEs production and oxidative stress. In hyperglycemic conditions, proteins related to mitochondrial respiratory chain proteins come to be more glycated, and mitochondrial deoxyribonucleic acid (DNA) gets damaged, leading to a self-perpetuating loop of oxidative stress and AGE construction [10].

Several preclinical and clinical investigations revealed that interventions for AGEs could help to reduce and prevent diabetic vascular complications [11]. Based on the pivotal role of AGEs in establishing oxidative stress and vascular disease, in this cross-sectional study we planned to investigate the association between AGEs levels and ABC goals of diabetes control. This may enhance the monitoring of diabetic patients and selection of more effective therapeutic approaches.

## Methods

### Study design

This cross-sectional study was prospectively conducted from January 2019 to December 2020. The study was carried out in the diabetes clinic of the Vali-Asr Hospital, affiliated with the Tehran University of Medical Science. The sample size was estimated by a statistic advisor by the following formula and considering a 5% margin of error, level of confidence of 95% (z = 1.96), d (precision) of 0.06 and P (expected prevalence of 0.5) which resulted in a sample size of 267. Considering the probable drop-out of 20%, 320 participants were proposed of which 23 patients declined to enroll in the study.

$$ {\text{N}} = \left( {{\text{Z 1}} - \alpha /{2}} \right)^{{2}} \, \times \,{{{\text{P }}\left( {{1} - {\text{P}}} \right)} \mathord{\left/ {\vphantom {{{\text{P }}\left( {{1} - {\text{P}}} \right)} {{\text{d}}^{{2}} }}} \right. \kern-\nulldelimiterspace} {{\text{d}}^{{2}} }} $$

Accordingly, a final sample size of 293 patients was included. Included patients were previously diagnosed with T2D, who were referred to the outpatient diabetes clinic for routine follow-up.

### Inclusion and exclusion criteria

All of the following inclusion and exclusion criteria were met by patients who enrolled in the research.

Inclusion criteria were defined as patients who:

1. Diagnosed with T2D for at least one year according to American diabetes association criteria [12]; and
2. Had complete demographic and previous clinical data in medical records.

Exclusion criteria were defined as patients who:

1. Were not satisfied to participate in this research;
2. Had known history of acute and/or chronic infection, chronic diseases, gestational diabetes mellitus, psychiatric illness, malignancies, pregnancy, end-stage renal disease, or blood transfusion in the last 3 months; and
3. Current use of cytotoxic, glucocorticoids, or immunosuppressive agents

### Data collection methods

A specialized endocrinologist visited T2D patients and trained investigators carried out an interview based on a standard questionnaire. Following demographic and clinical characteristics were recorded; sex, age, duration of diabetes, smoking status, alcohol intake, history of any diseases, anti-diabetic agent use, and any medication use.

Blood pressure, height, and weight were measured from all study participants. The 24-h urine samples were collected by patients to measure proteinuria. Fasting blood samples (10 cc) were taken from all individuals and were stored at – 80 °C until the testing time. Subsequently, fasting blood sugar (FBS), lipid profile, HbA1C level, creatinine, and AGEs levels were measured. Serum lipid concentrations (cholesterol, high-density lipoprotein [HDL], LDL, and triglycerides) were measured using direct enzymatic methods (Parsazmun, Karaj, Iran). FBS was determined using enzymatic calorimetric methods by the glucose oxidase test. HbA1C level was determined using high-performance liquid chromatography (A1C, DS5 Pink kit; Drew, Marseille, France). Urinary albumin concentrations were measured by an immunoturbidimetric commercial kit (Randox, Antrim, UK). AGEs levels were measured by the spectrophotometry method (FLUO star OPTIMA, BMG, Germany) described by Kalousová et al. The intra- and inter-assay analytical coefficient of variation were determined as 5.1% and 7.9%, respectively. [13]. The blood samples obtained from the patients were diluted to the ratio of 1 to 50 in peripheral blood smear pH 7.4. Fluorescence intensity was assessed at 350 nm excitation and 440 nm emission and indicated as the proportion of fluorescence emission expressed as percentages.

### Statistical analyses

To evaluate the normality of the data, the Kolmogorov–Smirnov test was used. Continuous data are shown as mean ± standard deviation (SD), and categorical variables are expressed as percentages. The Chi-square test was employed to investigate the association between categorical variables. To assess the mean differences between the two groups, the independent t-test was performed. Additionally, one-way ANOVA was applied to assess if there is any significant difference between means of more than two groups. AGEs concentration was analyzed to build the Receiver Operating Characteristics (ROC) curve and to define the optimum cut-off value, sensitivity, and specificity of serum AGEs to predict ABC diabetic goal achievement. Multivariable logistic regression examined the adjusted odds ratio of AGEs levels to predict ABC goal achievement of diabetes. All statistical analyses were conducted with IBM SPSS software, version 22.0 (SPSS Inc., Chicago, Illinois, USA), with a two-tailed P < 0.05 regarded as statistically significant.

### Ethics

The Research Ethics Committee of The Tehran University of Medical Sciences authorized the study protocol (ethical approval code: IR.TUMS.IKHC.1399.265). After approval, the study was initiated. Participants had been explained about the project and everyone had given written informed consent to participate. The participants' identities and records are kept privately. The patient’s treatment and pharmacological intervention would not change due to this research. This research has been conducted in agreement with the Declaration of Helsinki (2000) of the World Medical Association [14].

### Definitions

#### Treatment goals

According to the 2015 American diabetes association guidelines, the ABC goals for diabetes treatment were defined as HbA1c < 7.0%, LDL < 100 mg/dL, and BP < 140/90 mmHg (6).

#### Smoking cigarettes

Study participants who had the habit of smoking during the study period. More than one cigarette every day for at least six months was considered smoking [15].

#### Fasting blood glucose

FBS ≥ 126 and FBS < 126 were defined as poor glycemic control and controlled FBS levels, respectively.

#### Blood pressure

Hypertension was regarded as taking antihypertensive drugs or systolic blood pressure (SBP) ≥ 140 mmHg, and/or diastolic blood pressure (DBP) ≥ 90 mmHg quantified by automatic BP monitors in a sitting position [16].

#### Different treatments for diabetes

(1) oral use of anti-diabetic drugs, (2) Insulin, and (3) Both oral and insulin.

#### HbA1c

we defined HbA1c ≥ 9% as poor glycemic control, HbA1c 7–9% as sub-optimal and HbA1c < 7% as optimal glycemic control.

#### Triglycerides

amounts less than 150 mg/dL were considered normal and amounts equal to or more than that were defined as high triglycerides.

#### LDL and HDL and cholesterol

Amounts lower than 100 mg/dL for LDL and higher or equal to 40 mg/dL in males and more than 50 mg/dL in females for HDL and below 200 mg/dL for cholesterol were considered optimal control.

#### Anthropometric measurements

Using stretch-resistant tape, the midpoint between the bottom border of the last palpable rib and the superior margin of the iliac crest, was used to measure the circumference of the waist; the waist circumference greater than 88 cm for women and 102 cm for men was then regarded as central obesity.

#### BMI (body mass index)

Based on the definition of the World Health Organization, BMI is defined as dividing a person's weight (Kg) divided by height in meters squared (Kg/m^2^). In the categorization, below 25 is the optimal number, over 25 is considered overweight, and over 30 is obese.

#### Microalbuminuria

It was defined as between 30 and 300 mg/day of urine albumin excretion.

## Results

This research involved 293 participants with T2D. In total, about 60.4% (n = 177) were females and the others were male 39.6% (n = 116). As shown in Table 1, the majority of adults were aged 40–60 years old (60.8%), and 31.7% were equal to or above 60 years old. The average study population was 55.09 years old. The mean duration of diabetes among all patients was 7.65 years. Patients were mostly using just oral drugs to control their diabetes (61.4%). Besides, about 24.6% were on oral anti-diabetes drugs plus insulin at the same time. The prevalence of smoking was about 4.1%. In terms of BMI, 39.6% were normal weight and 38.6% were overweight, and the mean BMI was 27.37 kg/m^2^.

**Table 1:** Baseline characteristics of the patients

Variable	Frequency (%)	Mean ± SD	AGEs (Mean ± SD)	P-value
Age group (years)				
≥ 40	22 (7.5%)	55.09 ± 10.00	71.66 ± 12.23	0.428
40–60	178 (60.8%)		75.40 ± 11.73	
≤ 60	93 (31.7%)		74.71 ± 14.70	
BMI (kg/m^2^)				
< 25	64 (21.8%)	27.38 ± 3.95	77.79 ± 11.89	0.016*
25–30	116 (39.6%)		75.78 ± 12.45	
≥ 30	113 (38.6%)		72.36 ± 13.22	
Central obesity				
No	131 (44.7)	NA	75.61 ± 12.31	0.725
Yes	162 (55.3)		75.12 ± 13.18	
Smoking				
No	281 (95.9%)	NA	74.47 ± 12.74	0.004*
Yes	12 (4.1%)		85.14 ± 9.23	
FBS (mg/dL)				
< 126	26 (8.9%)	186.05 ± 61.19	71.88 ± 13.5	0.207
≥ 126	267 (91.1%)		75.19 ± 12.6	
HbA1C (%)				
< 7%	86 (29.4%)	8.18 ± 1.87	73.55 ± 12	0.323
7–9%	126 (43%)		74.78 ± 13.4	
≥ 9%	81 (27.6%)		76.52 ± 12.4	
SBP (mmHg)				
< 140	219 (74.7%)	125.73 ± 19.79	74.20 ± 12.3	0.107
≥ 140	74 (25.3%)		76.97 ± 13.8	
DBP (mmHg)				
< 90	240 (81.9%)	77.82 ± 10.56	74.10 ± 12.2	0.045*
≥ 90	53 (18.1%)		78.52 ± 14.6	
Therapeutic agent				
OHA	180 (61.4%)	NA	74.60 ± 12.18	0.326
Insulin	41 (14%)		76.21 ± 14.44	
Insulin + OHA	72 (24.6%)		74.93 ± 13.39	
Cholesterol (mg/dL)				
< 200	177 (60.4%)	190.42 ± 42.25	74.38 ± 12.8	0.389
≥ 200	116 (39.6%)		75.70 ± 12.6	
Triglycerides (mg/dL)				
< 150	149 (50.9%)	166.84 ± 80.36	75.04 ± 12.5	0.849
≥ 150	144 (49.1%)		75.75 ± 13.0	
LDL (mg/dL)				
< 100	114 (38.9%)	111.13 ± 33.44	74.13 ± 13.0	0.411
≥ 100	179 (61.1%)		75.39 ± 12.5	
HDL (mg/dL)				
< 40	82 (28%)	45.38 ± 9.61	77.34 ± 13.3	0.042*
≥ 40	211 (72%)		73.95 ± 12.4	

AGEs, advanced glycation end-products; BMI; Body mass index; FBS; Fasting blood sugar; HbA1C, hemoglobin A1c; SBP, Systolic blood pressure; DBP, Diastolic blood pressure; OHA, Oral hypoglycemic agents; LDL, low-density lipoprotein; HDL, high-density lipoprotein

*P < 0.05

The average FBS was 186.05 mg/dL. The majority of patients (91.1%) had high FBS (≥ 126 mg/dL). Their mean HbA1C level was 8.18%. Notably, about 43% and 27.6% had HbA1C between 7–9% and ≥ 9%, respectively. The mean (± SD) of SBP and DBP were 125 ± 19.7 mmHg and 77.8 ± 10 mmHg, respectively. The mean (± SD) of HDL, LDL, and TG were 45 ± 9.6 mg/dL, 111 ± 33 mg/dL, and 166.8 ± 80 mg/dL, respectively. The level of TC was 190 ± 42 mg/dL. The mean AGEs level was 74.91 ± 12.78%.

Furthermore, we investigated if there is any significant association between AGEs levels and other variables. The findings revealed a strong correlation between AGE levels and BMI (P-value 0.016), DBP (P-value = 0.045), HDL (P-value = 0.042), and cigarette smoking (P-value = 0.004). Additionally, the AGEs level was significantly associated with the presence of microvascular diseases (P-value = 0.001) and retinopathy (P-value = 0.001). No association between the AGEs levels and the presence of neuropathy, CVD, and microalbuminuria was found (Table 2).

**Table 2:** Association between AGEs level and diabetic complications

Variable	N (%)	AGEs level (%)	P-value
Microvascular complications			
No	174 (59.4)	70.41 ± 11.70	< 0.001*
Yes	119 (40.6)	81.48 ± 11.41	
Retinopathy			
No	230 (78.5)	71.98 ± 11.46	< 0.001*
Yes	63 (21.5)	85.60 ± 11.66	
Neuropathy			
No	196 (66.9)	75.20 ± 12.7	0.540
Yes	96 (32.8)	74.22 ± 13.1	
Microalbuminuria			
No	209 (71.3)	74.62 ± 11.5	0.720
Yes	81 (27.6)	75.30 ± 15.5	
CVD			
No	236 (80.5)	75.25 ± 12.6	0.421
Yes	57 (19.5)	73.48 ± 15.4	

AGEs, advanced glycation end-products; CVD, cardiovascular disease

*P < 0.05

Patients were categorized into four groups based on ABC goal achievement. A total of 67 patients (22.9%) had not achieved any of the ABC goals. More significantly, 108 people (36.9%) had achieved only one of the ABC goals. Ninety-three patients (31.7%) had reached a couple of goals and only 25 patients (8.5%) had successfully achieved all three ABC goals.

**Fig. 1:**
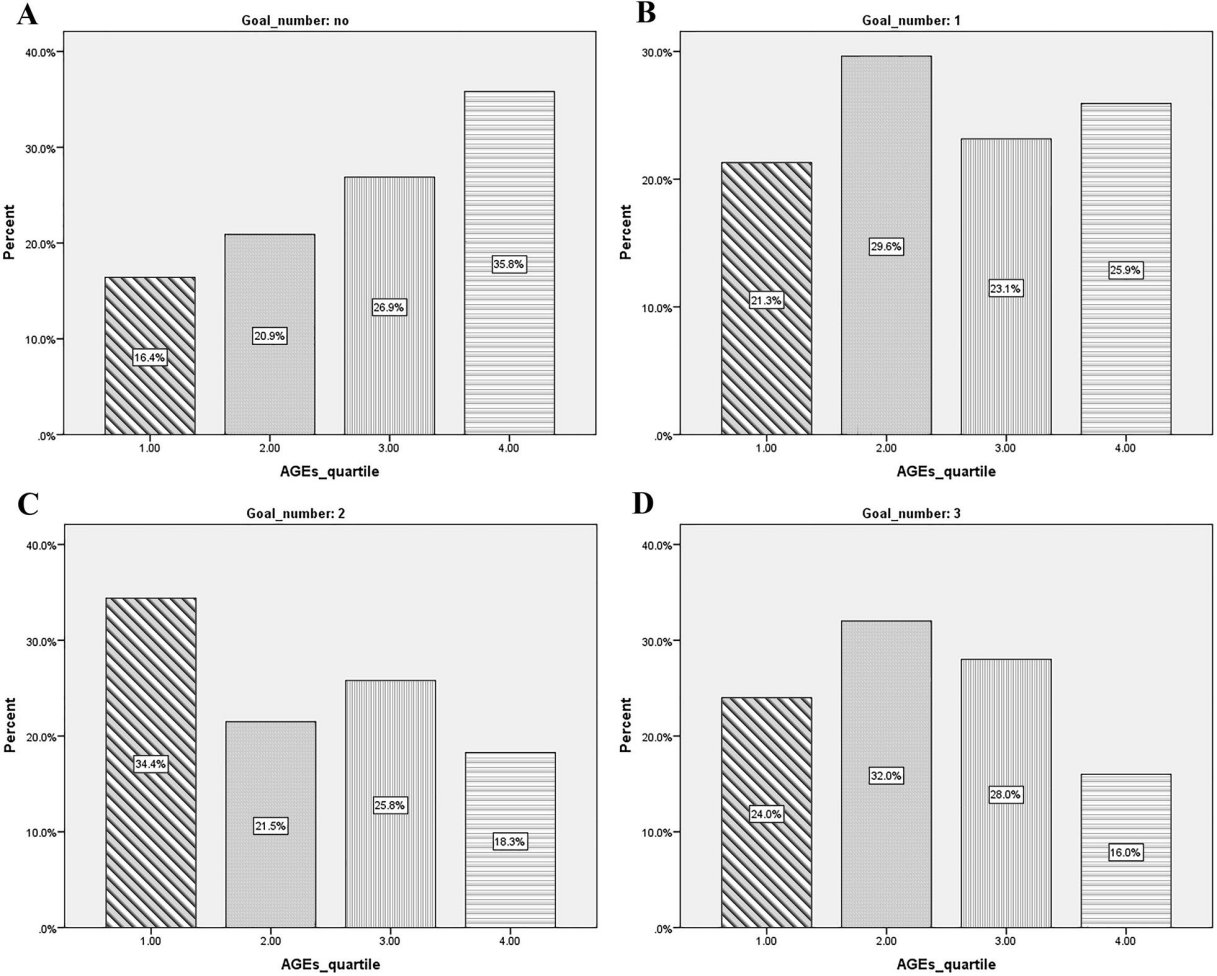
Segmentation of AGEs into four quartiles and comparing it with diabetic patients who: **A** have not reached any of ABC goals, **B** have reached one of the ABC goals, **C** have reached a couple of ABC goals, and **D** have reached all of the ABC goals

As shown in Fig. 1a, a large percentage of patients (35.8%) whose AGEs were in the highest quartile, had not reached any of the ABC goals. Patients who had reached a couple of ABC goals (Fig. 1c), were mostly (34.4%) placed in the first quartile of AGEs levels, which means they had a lower rate of AGE among all diabetic patients. Moreover, based on Fig. 1d, a much lower percentage (16%) of those patients who had reached all three goals of ABC, was placed in the fourth (highest) quartile of the AGEs level.

**Fig. 2:**
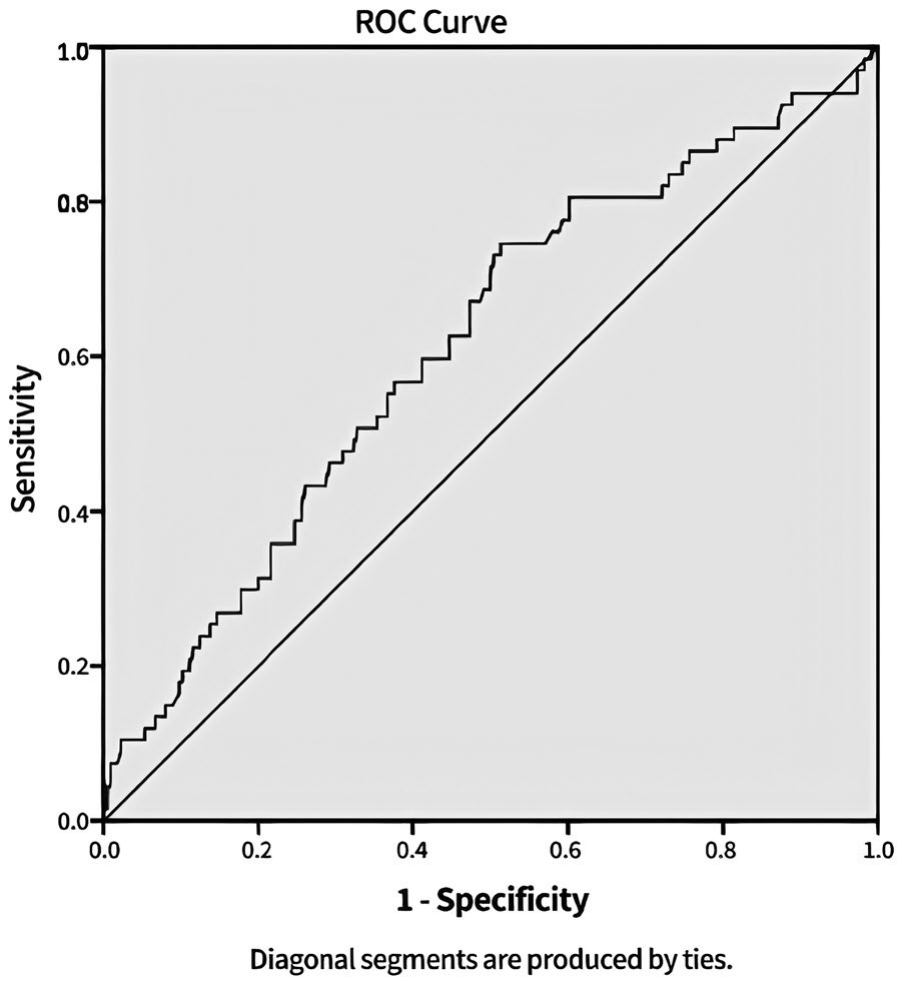
The performance of AGEs levels as a predictor of achieving ABC treatments goals

As a result, we hypothesized that the AGEs level may be a predictive factor for achieving the ABC goals. AUC-ROC analysis results indicate an AUC of 0.62 (95% CI 0.538–0.692), a sensitivity of 67%, and a specificity of 53%. The measured cut-off was 73.9 (Fig. 2).

Afterward, we investigated the association between ABC goal achievement of diabetes, and clinical and demographic characteristics of patients (Table 3). The findings revealed that there is a correlation between ABC goal achievement and the patient's age (P-value = 0.003), FBS (P-value = 0.002), and total cholesterol (P-value = 0.001) in addition to factors related to ABC goal achievements (HbA1C [P-value = 0.001], DBP [P-value = 0.001], SBP [P-value = 0.001], LDL [P-value = 0.001]) in multiple variable logistic regression analysis.

**Table 3:** The association between ABC goal achievement of diabetes, and clinical and demographic characteristics of patients

Variables	Number of ABC goals achieved				
	No	One	Two	Three	P-value
Age (years)	58.98 ± 9.3	54.39 ± 9.9	53.99 ± 9.7	52.44 ± 10.2	0.003*
Sex					
Female	19.2%	36.7%	33.3%	10.7%	0.148
Male	28.4%	37.1%	29.3%	5.2%	
Duration of T2D (years)	8.46 ± 5.9	6.93 ± 5.6	7.72 ± 6.4	6.12 ± 4.5	0.234
FBS (mg/dL)	197.9 ± 63	194.5 ± 67	173.9 ± 55	156.8 ± 32	0.002*
HbA1C (%)	8.8 ± 2.0	8.4 ± 1.3	7.7 ± 1.8	6.2 ± 0.5	0.001*
SBP (mmHg)	133.94 ± 21.9	129.36 ± 20.6	119.26 ± 14.7	112.12 ± 10.5	0.001*
DBP (mmHg)	80.79 ± 10.7	79.45 ± 10.4	74.94 ± 10.3	73.48 ± 7.1	0.001*
Cholesterol (mg/dL)	210.26 ± 39	198.41 ± 43	175.65 ± 36	157.56 ± 27	0.001*
Triglycerides (mg/dL)	171.53 ± 72	168.60 ± 82	161.61 ± 79	166.04 ± 98	0.880
LDL (mg/dL)	138.98 ± 25	112.64 ± 31	97.39 ± 29	81 ± 13	0.001*
HDL (mg/dL)	44.75 ± 9	45.39 ± 9	45.89 ± 10	45.08 ± 9	0.904
BMI (kg/m^2^)	26.56 ± 3.6	27.37 ± 3.9	28 ± 4.0	27.24 ± 3.8	0.155
AGEs (%)	78.42 ± 13.1	75.25 ± 12.9	72.17 ± 12.3	74.11 ± 10.4	0.023
Albuminuria (mg/day)	78.67 ± 15	74.86 ± 24	84.20 ± 28	78.38 ± 20	0.523
Treatment					
OHA	24.4%	36.7%	28.3%	10.6%	0.056
Ins	22%	51.2%	26.8%	0.0%	
OHA + Ins	19.4%	29.2%	43.1%	8.3%	

T2D, Type 2 diabetes mellitus; FBS, Fasting blood sugar; HbA1C, hemoglobin A1c; SBP, Systolic blood pressure; DBP, Diastolic blood pressure; LDL, low-density lipoprotein; HDL, high-density lipoprotein; BMI; Body mass index; AGEs, advanced glycation end-products; OHA, Oral hypoglycemic agents; Ins, Insulin

*P < 0.05

AGEs level was an independent predictor of no achievement of all three ABC goals after adjustment for age, sex, BMI, duration of diabetes, cardiovascular disease, and microvascular complications (OR: 2.29, 95% CI 1.02–5.18, P value:0.04).

## Discussion

In the current cross-sectional study, we explored the possible relationship of AGEs levels with clinical characteristics, macro- and microvascular complications of diabetic patients, and ABC goal achievement.

In the current study, it has been specified that smoker patients and those with higher DBP and lower HDL have statistically higher levels of serum AGEs concentration. Similarly, in the study by Indyk et al., there was a negative correlation between HDL level and concentration of melibiose-derived glycation product (MAGE) (r =  − 0.4220, P-value = 0.0093), which is a new class of AGEs, while there was no significant relationship among AGEs and LDL or total cholesterol [17]. Chang et al. introduced AGEs levels as a marker for poor lipid profile and atherosclerosis in diabetic individuals, with an even stronger association than FBS or HBA1c levels [18]. Previously, it has been shown that compared to non-smokers, AGEs accumulate in the tissues of smokers at higher levels, irrespective of diabetes [19]. Reactive glycation products present in cigarette smoke have been shown to promote AGE buildup in smokers' blood and tissues [20]. Another study indicated that AGEs were related to the pathophysiology of obesity [21]. We found a significant relationship between BMI and AGEs levels (P-value = 016). Remarkably, normal weight T2D patients (BMI < 25 kg/m^2^), had higher mean serum AGEs levels compared to overweight and obese T2D patients. Earlier studies reported a similar association in both diabetic and non-diabetic individuals [21, 22]. In the same way, Gaens et al. reported that waist circumference is negatively associated with plasma concentration of CML (*N* ^ε^-carboxymethyl lysine), which is a circulating AGEs (standardized regression coefficient [β] = − 0.357 [95% CI − 0.414; − 0.301], P < 0.001) [23]. At first glance, it seems paradoxical but could be rationalized by a concept that proposes decreased plasma AGEs levels might be attributed to AGEs being predominantly deposited in adipose tissue [24].

Because of their long residence duration in the human body, AGEs are currently thought to be a possible diagnostic marker. Monitoring of AGEs levels may enable the determination of disease progression, and therapeutic efficacy [17].

We indicated a relationship between elevated AGEs serum concentration and the occurrence of any microvascular complication (P-value ≤ 0.001), particularly retinopathy (P-value ≤ 0.001). Although in the cases of neuropathy and nephropathy, the AGEs levels were higher, the relationship was not statistically significant. This finding is different from previous studies which have demonstrated that AGEs or their receptor (RAGE) levels were significantly associated with diabetic neuropathy [25] and nephropathy [26, 27].

AGEs contribute to diabetes mellitus macro- and microvascular complications through adduct formation. Adducts form in basement membranes and cause changes in extracellular matrix proteins like elastin and collagen [28]. Moreover, it is suggested that AGEs, through interaction with RAGE, may result in the inflammatory process, oxidative stress, as well as development of clots and calcification in the arteries. As a result, AGEs are linked to blood vessel aging and damage. These findings imply that both AGEs and RAGEs are potential therapeutic targets to avoid diabetes-related vascular complications [17, 29, 30].

The possible role of AGEs in diabetic retinopathy is initiated by AGEs binding to RAGE that triggers intracellular signaling cascades. Pro-inflammatory cytokines may be produced as a result of this mechanism and proangiogenic mediates that cause inflammation, pericyte apoptosis, angiogenesis, endothelial dysfunction, and lastly, blood-retinal barrier breakdown. These events cause damage to vessels and neurons of the retina [31].

High AGEs levels are also reported to be related to poor diabetes control in terms of HbA1C [32, 33], lipid profile [18, 34], and blood pressure [33]. The current study has revealed that achieving the ABC goal is statistically associated with AGEs levels (P value = 0.023). It was also shown that diabetic patients with AGEs levels above 73.9% were 2.2 times more likely to achieve none of ABC treatment goals (95% CI 1.107–3.616). The result remains significant after adjustment of possible confounders like age, sex, and BMI. In a prospective cohort study of 781 individuals, it was observed that individuals reported that the average level of AGEs in normoglycemic individuals is about 246 µU/mL in baseline samples, while those with AGEs concentration equal to or higher than 450 µU/mL had about tenfold greater risk to develop T2D, 7 years later [35].

The basic limitation of the current investigation is the use of cross-sectional information, which prohibits us from establishing causal associations. Another limitation is that we did not investigate the role of antihypertensive and lipid-lowering drugs in the ABC treatment goals. It is worth mentioning that our proposed AGEs assay did not include measurement of melibiose-derived AGE (MAGE), which is produced from melibiose during in vitro glycation of proteins and protein-free amino acids in anhydrous conditions [36]. Furthermore, some factors like physical activity and diet are possible contributors to AGEs and diabetes complications, which have not been measured and adjusted [37]. Future multi-center research may address these limitations.

## Conclusion

In conclusion, current research revealed the relationship between AGEs and ABC goal achievement, and microvascular diabetic complications. Higher levels of serum AGEs were detected in older age, smoking patients and those with higher DBP, lower HDL levels, lower BMI and retinopathy. Moreover, diabetic patients with high AGEs levels were more likely to fail to achieve ABC treatment goals.

## Data Availability

The datasets used and/or analyzed during the current study are available from the corresponding author upon reasonable request.

## Abbreviations

AGEs: Advanced glycation end products
ABC: Hemoglobin A1C, blood pressure, and low-density lipoprotein cholesterol
LDL: Lipoprotein cholesterol
T2D: Type 2 diabetes mellitus
BMI: Body mass index
CVD: Cardiovascular disease
BP: Blood pressure
HbA1C: Hemoglobin A1C
RAGE: Receptor for advanced glycation end-product
DNA: Deoxyribonucleic acid
FBS: Fasting blood sugar
SD: Standard deviation
ROC: Receiver operating characteristics
SBP: Systolic blood pressure
DBP: Diastolic blood pressure
HDL: High-density lipoprotein
OHA: Oral hypoglycemic agents,
MAGE: Melibiose-derived AGE
